# A Novel Salt-Bridge Electroflocculation Technology for Harvesting Microalgae

**DOI:** 10.3389/fbioe.2022.902524

**Published:** 2022-06-17

**Authors:** Yuyong Hou, Chenfeng Liu, Zhiyong Liu, Tong Han, Nahui Hao, Zhile Guo, Weijie Wang, Shulin Chen, Lei Zhao, Maliheh Safavi, Xiang Ji, Fangjian Chen

**Affiliations:** ^1^ Key Laboratory of Systems Microbial Biotechnology, Tianjin Institute of Industrial Biotechnology, Chinese Academy of Sciences, Tianjin, China; ^2^ College of Life Sciences, Inner Mongolia Agricultural University, Hohhot, China; ^3^ National Center of Technology Innovation for Synthetic Biology, Tianjin, China; ^4^ College of Life Science, North China University of Science and Technology, Tangshan, China; ^5^ Department of Biotechnology, Iranian Research Organization for Science and Technology, Tehran, Iran

**Keywords:** microalgae, Nannochloropsis oculata, harvesting, salt-bridge electroflocculation, recovery efficiency

## Abstract

Microalgae biomass, as a promising alternative feedstock, can be refined into biodiesel, pharmaceutical, and food productions. However, the harvesting process for quality biomass still remains a main bottleneck due to its high energy demand. In this study, a novel technique integrating alkali-induced flocculation and electrolysis, named salt-bridge electroflocculation (SBEF) with non-sacrificial carbon electrodes is developed to promote recovery efficiency and cost savings. The results show that the energy consumption decreased to 1.50 Wh/g biomass with a high harvesting efficiency of 90.4% under 300 mA in 45 min. The mean particle size of algae flocs increased 3.85-fold from 2.75 to 10.59 µm, which was convenient to the follow-up processing. Another major advantage of this method is that the salt-bridge firmly prevented cells being destroyed by the anode’s oxidation and did not bring any external contaminants to algal biomass and flocculated medium, which conquered the technical defects in electro-flocculation. The proposed SBEF technology could be used as a low cost process for efficient microalgae harvest with high quality biomass.

## 1 Introduction

Microalgae, an autotrophic microbe which can be cultured in stress conditions and convert CO_2_ to various intracellular metabolites is considered as the renewable source of biofuel, nutraceutical, pharmaceutical, food, and animal feed (Borugadda and Goud, 2012; Chen et al., 2018; Li et al., 2021). However, low cell density and small cell size of photoautotrophic microalgae make the harvesting process a challenge in the microalgae industry. It was reported that the harvesting process accounts for 20–30% of the total cost of microalgae biofuels (Danquah et al., 2009; Uduman et al., 2010a). Thus, searching for economical and efficient methods for microalgae harvesting is critical for microalgae’s commercial application.

Currently, there are some common ways for microalgae harvesting, such as centrifugation, filtration, flocculation, and self-sedimentation (Liu et al., 2017). Among these techniques, centrifugation possesses the high energy consumption with ∼8 Wh/g biomass because of the dilute cell density of culture (generally lower than 2 g/L) (Sukenik et al., 1988; Molina Grima et al., 2003; Brentner et al., 2011). For membrane flocculation, a small diameter cell easily results in membrane block, the clogged cell wall can be broken by the pressure, the intracellular substance is useful to breed microorganism which leads to algae contamination (Gifuni et al., 2020). Self-sedimentation is time-consuming and results in a low concentration of the algal cake (Hapońska et al., 2018). Generally, the high cost of cell harvesting with those methods is a deterrent. Interestingly, flocculation techniques with the ability to concentrate the pending treated volume (more than ten folds) and enlarge the particle size of algal flocs seem to be convenient for downstream operation and have been regarded as the candidate for microalgae harvest (Uduman et al., 2011; Şirin et al., 2012).

Nevertheless, technology gaps of the flocculation process still exist in these reported techniques. Inorganic flocculants (ferric and aluminium sulfate) bring contaminations to the algae biomass and flocculated medium (Papazi et al., 2010; Wang et al., 2021). In contrast, organic flocculants demonstrate a high recovery efficiency of freshwater microalgae and leave few pollutants to the algae biomass, but only a part of them are efficient for marine microalgae harvest (Bilanovic et al., 1988). Besides, a high price of organic flocculants limits the availability in the industrial scale. Although electro-flocculation was reported as the new low-cost approach for cell harvesting, there are still some disadvantages. As a physical/chemical process, electro-flocculation generates positively charged precipitates to bind the negatively charged microalgae to form large microalgal flocs, which makes this technique efficient for harvesting small-size microalgae. On the other hand, sacrificial anodes’ corrosion (ferrous/aluminum) accounts for a considerable cost and brings contamination to the microalgae sludge and flocculated medium (ferric/aluminum ions’ accumulating). Meanwhile, in the process of the reaction, oxidants are generated and destroy the cell integrity as well as intracellular metabolites (Drees et al., 2003). Thus, the quality of microalgae biomass is difficult to be guaranteed. Therefore, a method with high recovery efficiency, low energy consumption, and high quality biomass for microalgae harvest is highly desirable.

In this study, the electrolytic cell was separated into independent cathode and anode chambers, which were filled with pending non-flocculent microalgae cells and sea water, respectively. Then, the two chambers were linked by a salt-bridge, graphite electrodes were used instead of metal electrodes (ferrum/aluminum). When the system was powered on, the oxidants were segregated into the anode chamber, electrons were transferred to the cathode chamber through the salt-bridge to proceed with the reduction reaction (hydrogen ion was catalyzed to hydrogen). The pH of the cathode chamber was thus increased gradually, and then induced cells flocculation with the culture medium containing metal cations. This article presents the test results of the salt-bridge electroflocculation (SBEF), including flocculation efficiency, energy consumption, and cell metabolite analysis. The mechanisms of this flocculation process are also described in detail. Upon further refinement, SBEF has provided a viable option for microalgae harvesting to make algal production more competitive.

## 2 Materials and Methods

### 2.1 Strain and Culture

The strain *Nannochloropsis oculata* (*N. oculata*) with high lipid productivity, came from the Culture Collection of Algae and Protozoa at the Scottish Marine Institute (Oban, Argyll, United Kingdom) and was difficult to harvest on account of its small cell size and low cell density (Guldhe et al., 2016; Ishika et al., 2021). The cells were cultivated in a 2 L Erlenmeyer flask containing 1.2 L f/2 medium under continuous illumination of 150 μmol m^−2^·s^−1^ with 2% filtered CO_2_ (v/v) at 25°C (Guillard and Ryther, 1962). The initial cell density was 0.15 g/L; the final biomass for SBEF experiments was about 1.4 g/L during 14 days cultivation.

### 2.2 SBEF System

**FIGURE 1:**
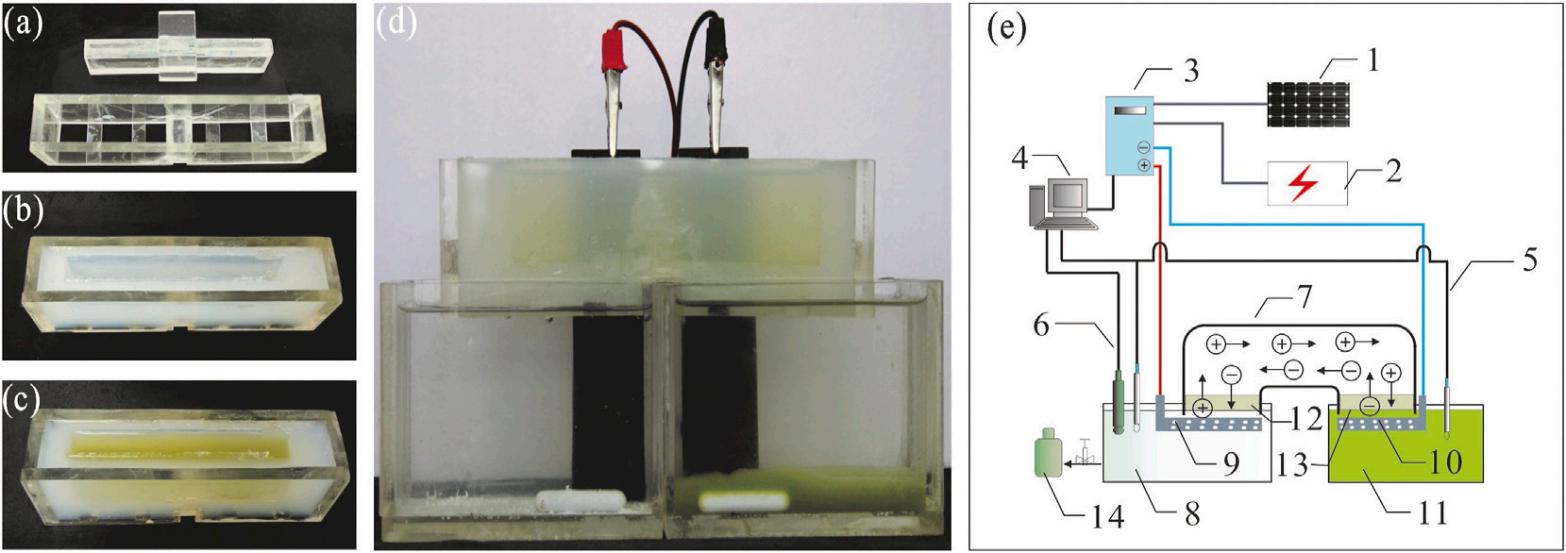
Schematic of salt-bridge electroflocculation (SBEF) for harvesting *N. oculata*: **(A–C)** tools to shape the salt-bridge; **(D)** apply SBEF to harvest *N. oculata* (stay for 20 min when finished); **(E)** a conceptual design for SBEF coupling solar cells. Legends in Figure 1E: 1, solar cells; 2, public power network; 3, regulator; 4, computer; 5, pH meters; 6, active chlorine meters; 7, salt-bridge; 8, anode chamber; 9, anode; 10, cathode; 11, algae pond; 12, agar layer or anode membrane; 13, agar layer or cathode membrane; and 14, bleach.

As shown in Figure 1, the structures of SBEF were designed specifically, including the salt-bridge, electrodes, and reaction chambers. The salt-bridge (Figure 1B) was shaped by a plexiglass shell (Figure 1A). The melting agar solution was poured into the plexiglass shell (agar:seawater = 1.5:100 in weight, the salinity of seawater was about 30‰), and a salt-bridge of ∼100 ml inner volume was shaped after cooling to room temperature. The ion exchange area of the salt-bridge was approximately 15 cm^2^ (3 × 5 cm), which directly contacted with the liquid interface of both anode electrolytes and the microalgae culture (Figure 1D). Both the anode and cathode (Figure 1D, tied by red and black wires, respectively) were made of graphite for avoiding corrosion. Solar energy was used in the conceptual design to further decrease the cost (Figure 1E).

During the SBEF process, 250 ml seawater and the microalgae culture were poured into the anode and cathode chambers, respectively (Figure 1D), meanwhile 100 ml algae suspension was poured into the salt-bridge channel (Figure 1C). Stirring at a gentle speed of 50 rpm was supplied by a magnetic stirrer (82–2 type, Shanghai Sile instrument Co., Ltd). When SBEF was finished, the treated culture from both the cathode chamber and salt-bridge channel (350 ml in total) were mixed, then, the recovery efficiency and microalgae pH were measured.

### 2.3 Studies Under Varying Conditions in SBEF

It is obvious that the main factors affecting the efficiency of SBEF include operation times, current intensity, biomass density, and salinity of the system. A preliminary study of high current intensity (450 mA) with operation time ranging from 10 to 50 min was carried out to assess the recovery, efficiency, and electrical energy consumption (EEC) of SBEF. Subsequently, to obtain the minimum EEC and maximum recovery efficiency (RE), the low current intensity of 150 and 300 mA with more complicated operation time (15, 30, 45, 60, 75, 90, 105, 120, 135, and 150 min) was performed. To further characterize SBEF, the initial microalgae biomass was then modified to 0.5-, 2-, and 3-fold of 1.4 g/L, marked as 0.5X, 2X, and 3X. For biomass variation studies, the cells were diluted with the microalgae supernatant for 0.5X and concentrated by hollow fiber microfiltration membrane devices (MOF-2b, 0.1 μm in aperture of separation, Tianjin Motimo Membrane Technology CO. Ltd.) for 2X and 3X, respectively. Meanwhile, the salinity of microalgae medium and salt-bridge with the following levels were also examined: 20, 30, 40, and 50‰, which were adjusted by distilled water or sea salt. The salinity of the anode chamber was regulated simultaneously, which was required to be consistent with the cathode chamber and salt-bridge.

### 2.4 Algae Analytical Methods

The recovery efficiency, RE ( ${\text{η}}_{\text{a}}$ ), and concentration factor (CF) were calculated as follows (Vandamme et al., 2011):

$$\mathrm{Recovery efficiency (}\eta_{a})=\frac{OD_{i}-OD_{f}}{OD_{i}}\times 100\%$$ (1)

$$\mathrm{Concentration fator}\left(\mathrm{CF}\right)=\;H_{total}/H{\;}_{flocs}$$ (2)

where *OD*_*i*_ is *OD*_*680*_ of the algal culture before SBEF, and *OD*_*f*_ is *OD*_*680*_ of the supernatant of the algae culture when poured into a 500 ml cylinder for 2 hours’ sedimentation; *H*_*total*_ is the total height of 350 ml algae suspension when poured into a 500 ml cylinder, and *H*_*flocs*_ is the height of the flocs layer in cylinder after 2 hours’ sedimentation of flocculated culture. The electrical energy consumption (in Wh/g biomass) of the SBEF process was calculated as Eq. 3 (Parmentier et al., 2020):

$$\mathrm{Electrical energy consumption}\left(\mathrm{EEC}\right)=\frac{UIT}{V\rho \eta_{a}}$$ (3)

where *U* is the operation voltage of SBEF (V), *I* is the current intensity (A), *T* is the operation time of SBEF process (h), *V* is the volume of the algae culture treated (0.35 L), ρ is the biomass density of the culture treated (g/L), and $\eta_{a}$ is the recovery efficiency that can be calculated by Eq. 1.

### 2.5 Focused Beam Reflectance Measurement of Flocs in SBEF Process

To evaluate the particle size distribution, the flocs of the microalgae that formed and settled down at the bottom of the cylinder was collected via emptying the supernatant medium and then measuring by FBRM (FBRM D600L, Mettler toledo) according to the previous study (Uduman et al., 2010b). Meanwhile, a sample culture without SBEF treatment was regarded as the control group.

### 2.6 Monitoring the Content of Flocculated Ions/Elements During SBEF Process

To reveal the mechanism of the SBEF process, the changes of the element contents in SBEF were measured. Microalgae sludge from SBEF and centrifugation were both dried by vacuum freezing dryer, and then identified by ICP-AES (IRIS Advantage ICAP, Thermo Electron Corporation) (Mayers et al., 2017). Briefly, 40 mg algae powder was poured into the hydrothermal synthesis reactor (Zhengzhou Boke Instrument Equipment Co. Ltd.) with 1 ml hydrogen peroxide solution (30%, V/V, GR) and 3 ml hydrogen nitrate (GR) to incubate for 30 min under 18°C condition. Then, the digested liquid was transferred to identify the content of key elements by ICP-AES according to China SN/T 2208-2008, including calcium (Ca), cobalt (Co), magnesium (Mg), ferrum (Fe), cuprum (Cu), manganese (Mn), molybdenum (Mo), silicon (Si), and zinc (Zn). Furthermore, the supernatant samples of the flocculated medium was collected by centrifugation in 8,000 g × 10 min, then used for magnesium and calcium concentration analyses based on China GB Standards of 15452-2009.

### 2.7 Measurement of Pigments, Protein, and Lipid Content

To further analyze whether the main microalgae intracellular metabolites were affected by SBEF, the contents of the pigments, proteins, and lipids from SBEF and centrifugation groups were detected, respectively.

For the pigments analysis, the cell samples were centrifuged at 8,000 rpm for 10 min and washed twice to wipe off the sediments that adhered to the cell wall by 50 mM EDTA·2Na solution. Then the pellets were homogenized via ultrasonic disruption (Sonics & Materials Inc., Newtown, CT, United States) with cold acetone. The chlorophyll and carotenoid content were calculated through spectrophotometry as described in (Macías-Sánchez et al., 2007).

Proteins were extracted according to our previous report (Chen et al., 2012). Briefly, the samples were treated by 0.5 M NaOH and boiled for 10 min, the supernatant was then centrifuged at 8,000 g × 10 min for protein measurement by using the Bradford assay (Bradford, 1976).

Chloroform–methanol method was used to extract the total lipid (Chen et al., 2012). About 40 mg biomass was mixed with 6 ml the chloroform–methanol (2:1, V/V) solution and vortexed extremely. After adding another 2 ml methanol and 3.6 ml 5% NaCl, the organic phase was collected at 8,000 g × 10 min. The chloroform layer was dried at 60°C with the protection of N_2_ in the pre-weighed test tube to achieve constant weight. The lipid content was calculated using the difference between the final and beginning weights of the tubes.

### 2.8 Statistical Analysis

All the experiments in this study were carried out thrice. Data are represented as the mean value of three independent replicates with standard deviation (error bars).

## 3 Results and Discussion

### 3.1 Effect of Variables on SBEF

#### 3.1.1 Effect of Operation Time on SBEF

**FIGURE 2:**
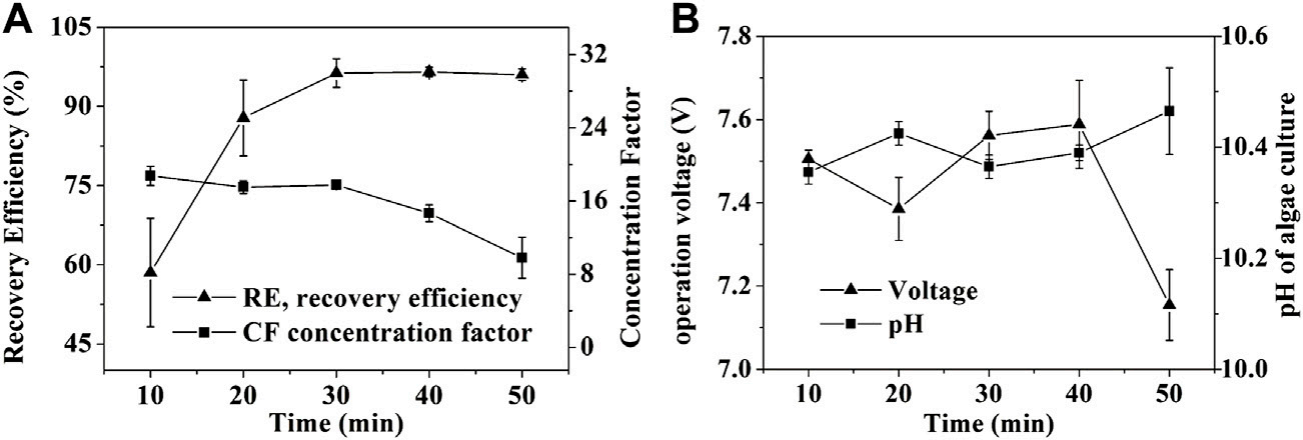
Effect of operation time on SBEF when harvesting *N. oculata* at 450 mA: **(A)** recovery efficiency and concentration factor; **(B)** operation voltage and pH of algae culture. Data were average ±standard deviation of three independent determinations.

**FIGURE 3:**
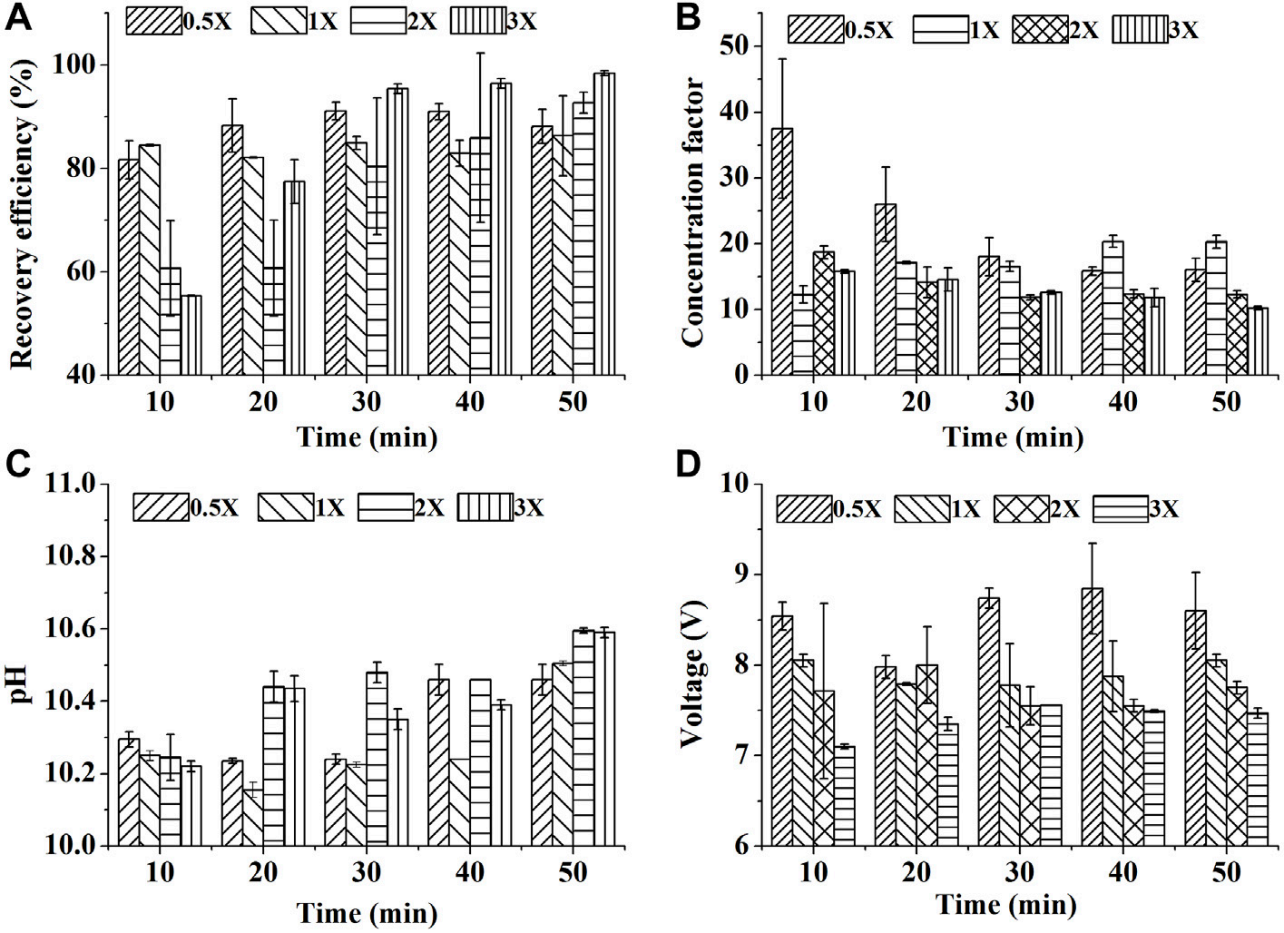
Effect of biomass density on SBEF when harvesting *N. oculata* at 450 mA: **(A)** recovery efficiency; **(B)** concentration factor; **(C)** pH of algae culture; **(D)** operation voltage; 1X stands for ∼1.4 g/L of the group in one-fold biomass density. Data were average ±standard deviation of three independent determinations.

**FIGURE 4:**
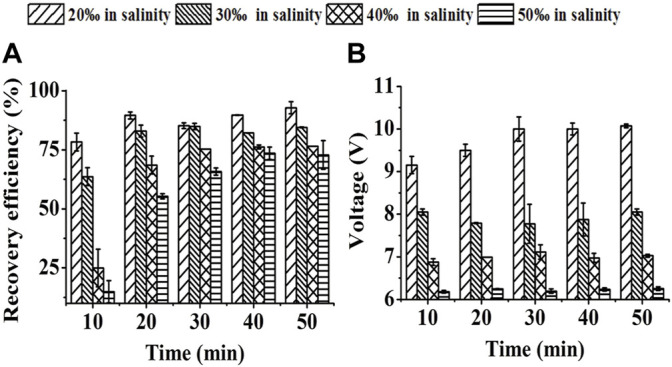
Effect of medium salinity on SBEF when harvesting *Nannochloropsis oculata* at 450 mA: **(A)** recovery efficiency; **(B)** operation voltage. Data were average ±standard deviation of three independent determinations.

In accordance with the hypothesis that the amount of induced flocculants is depended of operation time (Vandamme et al., 2011), the recovery efficiency of SBEF increased along with increasing time, but concentration factor (CF) decreased (Figure 2A). Similar results were observed in biomass and salinity studies (Figures 3A,B; Figure 4). The operational voltage was kept stabilized with a slight change, ranging from 7.38 to 7.58 during the electrolysis process (Figure 2B). As Eq. 3 shows, EEC of SBEF was affected dramatically by the operation time, thus the energy consumption increased from 1.96 to 5.70 Wh/g biomass when the system was prolonged to 50 min and yielded recovery efficiencies of 58.5%, 87.8%, 96.3%, 96.5% and 96.0%, for each 10-min interval, respectively (Table 1). In this study, although the reaction in the 30 and 40 min groups showed optimum RE (96.3% and 96.5%), the EEC was 1.4-fold and 1.87-fold higher than those in 20min with comparable RE (87.8%), respectively. It seems that the economic operation time of SBEF was 20 min, since recovery efficiency cannot increase obviously from the 20th min and the EEC kept at a low level (2.57 Wh/g biomass for 20 min), which is significantly lower than that in electrocoagulation with 9.16 Wh/g biomass for *Tetraselmis sp.* and 4.44 Wh/g biomass for *Chlorococcum sp* (Uduman et al., 2011)*.* Under this condition, the CF reached 18-fold (Figure 2A), which was obviously higher than the fold changes from the flocculation process induced by adding other flocculants like polyaluminum chloride (∼6.5), aluminum sulfate (∼7.2), and chitosan (∼8.9) (Şirin et al., 2012). Therefore, applying SBEF for harvest *N. oculata* may significantly reduce the volume of treated algal suspension and benefit the following processes such as centrifugation and filtration (Salim et al., 2012; Kim et al., 2013). Correspondingly, the EEC of SBEF was lower than tangential flow filtration (3.58 Wh/g biomass) and polymer flocculation (36.81 Wh/g biomass) (Uduman et al., 2011; Kim et al., 2012). Hence, these results indicate that SBEF is well-suited for harvesting *N. oculata*.

**TABLE 1:** Performance of SBEF on harvesting *N. oculata* at 450 mA.

Current intensity (mA)	Parameters	Treated time (min)				
		10	20	30	40	50
450	RE	58.5 ± 10.3	87.8 ± 7.2	96.3 ± 2.7	96.5 ± 0.9	96.0 ± 1.1
	V	7.5 ± 0.02	7.38 ± 0.07	7.56 ± 0.05	7.58 ± 0.10	7.15 ± 0.08
	Cc	75	150	225	300	375
	EEC	1.97	2.57	3.61	4.82	5.7

Note: RE, Recovery efficiency (%); V, Operation voltage (V); Cc, Charge consumption (mAh); EEC, Electrical energy consumption (Wh/g biomass). Data were average ±standard deviation of three independent determinations.

#### 3.1.2 Effect of Current Intensity on SBEF

With the increase of current intensity from 150 to 300 mA, the time to reach the maximum recovery efficiency was saved, which was decreased from 90 to 45 min (Supplementary Table S1). It was a coincidence with the Faraday’s laws of electrolysis that the amount of substance that was produced depended on the charge consumption (Liu et al., 2017). Current intensity of 450 mA with 30 min yields 225 mAh charge consumption, which was equaled to 150 mA with 90 min and 300 mA with 45 min (Table 1; Supplementary Table S1). With those conditions, the recovery efficiency remained almost unchanged. Different current intensities caused different applied potentials and affected the EEC (Eq. 3). Thus, the important aspect for SBEF should be optimized for maximum RE and minimum EEC. In this study, 450 mA of 40 min showed maximum RE (96.5%), however the EEC was 3.21-fold higher than 300 mA of 45 min. Meanwhile, the RE was comparable. As a result, the optimization condition of SBEF obtaining approved recovery efficiency was 300 mA for 45 min, yielded EEC of 1.50 Wh/g biomass and recovery efficiency of ∼90.4%, respectively. Compared to the low EEC technology of the electrochemical harvesting method that 1.76 Wh/g biomass with 1,000 mA applied current for *Ankistrodesmus falcatus*, the SBEF process still has the advantage of cost and well safety (Guldhe et al., 2016).

#### 3.1.3 Effect of Biomass Density on SBEF

Harvesting algae culture of high cell density was a bottleneck to the other types of flocculation (Zheng et al., 2012). The harvesting efficiency decreased from 95% in 0.4 g/L of biomass density to 80% in 2 g/L of biomass density with poly (γ-glutamic acid) addition. To evaluate whether SEBF is suitable for high cell density harvest with time saving, high current intensity of 450 mA with different biomass was used. Luckily, SBEF seems to overcome this technology challenge.

With increasing reaction time, the pH increased, then, the formed alkali-induced flocculant caused 98.5% RE in the 3X group within 50 min of treatment, which was higher than those in the0.5X, 1X, and 2X groups (Figures 3A,C). Even in the 30 min treatment, the RE of 3X was up to 95.5%. High RE resulted in more cell aggregation in the flocs’ layer and leaded to bigger volume, thus the CF could be decreased by the excess volume of the algal layer (Figure 3B). On the other hand, the low resistance loads caused by high cell density reduced the output–voltage swing (Figure 3D). Thus, based on Eq. 3, the EEC could also benefit from the system that the EEC of 0.5X team treated by SBEF for 30 min was approximately 8.81 Wh/g biomass, but 1.21 Wh/g biomass in 3X team (∼4.2 g/L in biomass density), which is much lower than centrifugation (65.34 Wh/g biomass) and 31.3% lower than the reported innovative electrochemical process (Guldhe et al., 2016). In brief, SBEF is suitable for harvesting algal culture in high biomass density.

#### 3.1.4 Effect of System Salinity on SBEF

As higher salinity conditions caused higher conductivity, the operation voltage thus gradually decreased when the salinity of this system increased from 2‰ to 5‰ with the constant-current used in SBEF (Figure 4B). However, a high ionic strength in the seawater medium led to a competitive combination with the cell wall which was expected to be only adsorbed by flocculant molecules (Sukenik et al., 1988; Schlesinger et al., 2012). Hence, the recovery efficiency with 92.8% in 2‰ salinity decreased to72.8% in 5‰ salinity with the 50 min treatment (Figure 4A), which was similar with the reports that the inhibition of recovery efficiency with higher medium salinity was extensive (Cheng et al., 2011; Zheng et al., 2012). Interestingly, the RE with 3‰ salinity which was suitable for various marine microalgae cultivations reached 84.9%. These results indicate that SBEF has the potential to harvest the cells cultured in marine conditions.

### 3.2 Mechanism Analysis of SBEF

To elucidate the mechanism of SBEF, two of the reactions described below were proposed in the cathode and anode chambers, respectively.

In the cathode chamber,

$$2H_{2}\mathrm{O=2}H^{+}\mathrm{+2O}H^{-}$$ (4)

$$2H^{+}\overset{\mathrm{+2e}}{\to }\;H_{2}↑$$ (5)

$$\mathrm{2O}H^{-}\mathrm{+ M}g^{\mathrm{2+}}\mathrm{=Mg(OH}{)}_{2}↓$$ (6)

In the anode chamber,

$$\mathrm{2O}H^{-}\overset{-2\text{e}}{\to }\;O_{2}↑+2H^{+}$$ (7)

$$\mathrm{2C}l^{-}\overset{\mathrm{-2e}}{\to }Cl_{2}↑$$ (8)

$$\mathrm{2C}l_{2}\mathrm{+2CaC}O_{3}\mathrm{=CaC}l_{2}\mathrm{+ Ca}{\left(\mathrm{ClO}\right)}_{2}\mathrm{+C}O_{2}↑$$ (9)

**FIGURE 5:**
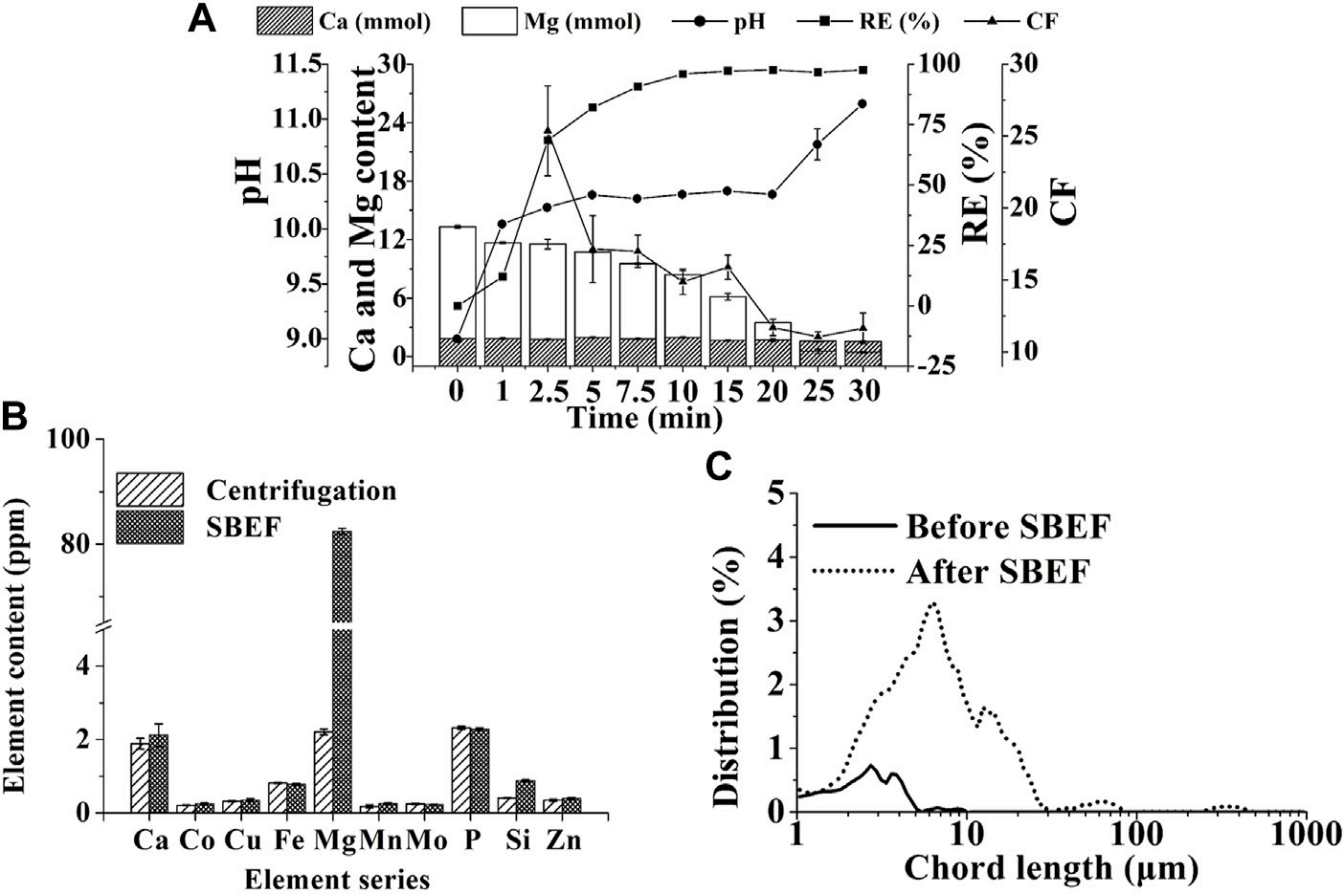
Flocculation mechanism of SBEF: **(A)** performance of SBEF with varied operation time (concentration of Ca^2+^ and Mg^2+^, pH of algae culture, recovery efficiency, and concentration factor); **(B)** the content of key elements in *N. oculata* biomass; **(C)** algae particle size distribution during the SBEF process. Data were average ±standard deviation of three independent determinations.

As shown in Eqs 4, 5, the pH of the cathode chamber in the algae pond (Figure 1E—11) was increased with hydrogen emission (Figures 2B, 3C). Thus, the flocculant, magnesium hydroxide (Eq. 6), was generated to result in microalgae flocculation (Vandamme et al., 2012). Therefore, the concentration of Mg^2+^ in the cathode chamber significantly decreased from 13.3 to 0.45 mM in 30 min (Eq. 6; Figure 5A). At the same time, Ca^2+^ content just decreased slightly (0.3 mM) in the algae supernatant and increased equivalently (0.23 mM) in the algal biomass (Figures 5A,B). The results indicated that magnesium hydroxide dominated cell flocculation, which was consistent with the previous study (García-Pérez et al., 2014). To verify the results, the Mg^2+^ contents in the algal biomass from centrifugation and SBEF were also compared. The Mg^2+^ content of flocculated algal powder is approximately 40-fold higher than those in the centrifugation group (Figure 5B). In contrast, the other elements including (Co, Cu, and Fe….) showed no significant differences (*p* > 0.05).

Considering that the main precipitation particles, Mg(OH)_2_, had a positive charge, the microalgae cells with negative charge were adhered to the pellets according to the electrostatic force by charge neutralization, then formed bigger flocs via adsorption bridging mechanism (Uduman et al., 2010b). The particle size of the microalgae cells distributed between 1 and 4 µm in untreated groups. For SBEF conditions, the flocs’ size was dominated in 10–100 µm. The mean size of the particles increased dramatically from 2.75 to 10.59 µm (Figure 5C), which could be benefited to the following processes in an economic and convenient way. Moreover, the pypocholoride that produced in the anode chamber could further offset the cost of SBEF. And the pypocholoride was preserved skillfully in this chamber due to the obstruction of the salt-bridge, which made up the defects of traditional electroflocculation without of any exogenous contaminations and cell oxidative damage (Drees et al., 2003). On the other hand, hypochlorite produced in the anode chamber could be used as disinfectants for microalgae bioreactor treatment and further reduce the cost of cell harvest.

Furthermore, traditional electroflocculation with the oxidation of metal electrodes results in electrode depletion and metal ion dissemination, thus the electrode and harvested biomass require periodic replacement and washing, respectively (Guldhe et al., 2016). In this study, SBEF technology with non-sacrificial carbon electrodes therefore remained competitive with other metallic electrode methods.

### 3.3 Effect of SBEF on Metabolites

The results showed that the lipid content of *N. oculata* harvested from centrifugation was 49.7% dry biomass, whereas that using SBEF was 47.3% dry biomass (Table 2). Similar results were obtained on protein, chlorophyll, and carotenoid content analyses (Table 2). Apparently, there are no significant differences on the metabolites between SBEF and centrifugation (*p* > 0.05). The quality of the microalgae product quality is ensured.

**TABLE 2:** Influence of SBEF on microalgae metabolites: lipids, proteins, chlorophyll, and carotenoids.

Items	Harvested by centrifugation	Harvested by SBEF
Lipid content (%)	49.7 ± 3.1	47.3 ± 3.8
Protein concentration (in alkaline extraction, mg/ml)	0.308 ± 0.020	0.314 ± 0.011
Chlorophyll concentration (in acetone extraction, μ g/ml)	2.174 ± 0.083	1.994 ± 0.093
Carotenoid concentration (in acetone extraction, μ g/ml)	1.325 ± 0.068	1.215 ± 0.079

Note: Data were average ±standard deviation of three independent determinations.

### 3.4 Economic Analysis

To further reduce the cost of SBEF, SBEF was designed to couple with solar cells (Figure 1E). The solar cell Hi-MO 5 m LR5-72HPH 555 M (LONGi Solar, 2,256 × 1,133 × 35 mm) with 545 W solar panel board power, can be used for 30 years, possesses 21.7% energy conversion efficiency, which is annually decreased by 0.55% per year. Considering that the annual mean sunshine duration is about 4 h/d in China, one solar cell can output 4,778 kWh electric energy. Based on the price of LR5-72HPH 555 M (1.88 RMB/W), the electricity price from a solar cell is 0.21 RMB/kWh by theoretical calculation, which is only 50% of the household electricity and 27.8% of the non-household electricity price in China. On the other hand, the Cl^−^ concentration is about 0.54 M in seawater. Based on the principle of seawater electrolysis, the max Cl_2_ content was calculated as 0.27 M, and with the addition of low-cost CaCO_3_, 0.135 M Ca(ClO)_2_ was obtained (Khan et al., 2021; Feng et al., 2022). It means than 1.5 Wh electric energy harvested 1 g *N oculata* biomass, could produce 0.15 M bleach. The price of CaCO_3_ is about 260 RMB/t, in contrast, the price of Ca(ClO)_2_ can reach 7,000 RMB/t (Alibaba). The byproduct, bleach, can produce economic benefits with 0.15 RMB/L seawater, which further decreases the cost of SBEF. Taking the EEC of centrifugation into consideration (∼8 Wh/g), the presented method possessed a high commercial value to instead centrifugation for a high quality microalgal biomass harvest, as the cost of SBEF was about 80% lower than centrifugation.

## 4 Conclusion

A novel electroflocualtion named salt-bridge electroflocculation (SBEF) was developed to harvest *Nannochloropsis oculata* with high recovery efficiency (>90%) and low energy consumption (1.50 Wh/g biomass). Meanwhile, SBEF did not bring any external flocculants to algal biomass and affect algae metabolites. The algal flocs from SBEF could be filtrated easily for its huge particle size (∼10.59 μm). The excellent characterization of SBEF for *N. oculata* could promote the development of the microalgae industry.

## Data Availability

The original contributions presented in the study are included in the article/Supplementary Material; further inquiries can be directed to the corresponding authors.
