# Supplementary material for: A Novel Salt-Bridge Electroflocculation Technology for Harvesting Microalgae
Source: Front Bioeng Biotechnol. 2022 Jun 17;10:902524. doi: 10.3389/fbioe.2022.902524 (PMC9247570; doi:10.3389/fbioe.2022.902524)
Supplement: Supplementary file 1 [file Table1.DOCX]

**Supplemental data**

**Table S1** Performance of SBEF on harvesting *N.* *oculata* at various current intensity (150 mA and 300 mA).

| Current inentsity | Parameters | Time (min) | | | | | | | | | |
| --- | --- | --- | --- | --- | --- | --- | --- | --- | --- | --- | --- |
|  |  | 15 | 30 | 45 | 60 | 75 | 90 | 105 | 120 | 135 | 150 |
| 150mA | RE | 2.4±1.1 | 4.5±0.4 | 29.1±3.8 | 70.2±2.0 | 73.8±15.4 | 89.1±1.0 | 90.0±1.1 | 89.6±0.6 | 81.9±1.6 | 79.2±1.2 |
|  | V | 4.44±0.01 | 4.44±0.01 | 4.44±0.01 | 4.46±0.01 | 4.47±0.01 | 4.48±0.01 | 4.47±0.01 | 4.48±0.01 | 4.49±0.01 | 4.48±0.01 |
|  | Cc | 37.5 | 75 | 112.5 | 150 | 187.5 | 225 | 262.5 | 300 | 337.5 | 375 |
|  | EEC | 14.16 | 15.1 | 3.5 | 1.94 | 2.32 | 2.31 | 2.66 | 3.06 | 3.78 | 4.33 |
| 300mA | RE | 8.8±2.9 | 78.1±0.7 | 90.4±0.2 | 90.6±0.6 | 89.8±0.2 | 91.4±0.14 | 89.0±2.8 | 91.2±1.1 | 91.1±0.9 | 90.6±1.6 |
|  | V | 5.99±0.01 | 5.93±0.06 | 5.92±0.01 | 5.91±0.01 | 5.91±0.01 | 5.88±0.02 | 5.87±0.01 | 5.86±0.01 | 5.87±0.01 | 5.8±0.02 |
|  | Cc | 75 | 150 | 225 | 300 | 375 | 450 | 525 | 600 | 675 | 750 |
|  | EEC | 10.42 | 2.32 | 1.5 | 2 | 2.52 | 2.95 | 3.53 | 3.93 | 4.44 | 4.9 |

Note: RE, Recovery efficiency (%); V, Operation voltage (V); Cc, Charge consumption (mAh); EEC, Electrical energy consumption (Wh/g biomass). Data were average ± standard deviation of three independent determinations.
